# Attitudes Toward Patient Safety Among Final-Year Medical Students and Interns at a Teaching Hospital in Kerala: A Cross-Sectional Study

**DOI:** 10.7759/cureus.73405

**Published:** 2024-11-10

**Authors:** Anuvinda Anil, Sreelakshmi Mohandas, Mufeeda Moidu, Shyama Karichery, Neha Jacob, Nandana Shanavas, Veena Pottekkattu, Arunima Chellappan, Fidah Nizar, Haritha Mohan

**Affiliations:** 1 Community Medicine, Amrita Institute of Medical Sciences, Amrita Vishwa Vidyapeetham, Kochi, IND

**Keywords:** attitude, interns, medical error, medical students, patient safety

## Abstract

Background: With the advancements in medicine, medical technologies, and diagnostics, patient safety is expected to be one of the priorities of modern medicine. However, in India, several medical error-related deaths have been recorded. Medical students today are the future healthcare providers, and hence, understanding their attitudes toward patient safety is crucial. The objective of this study was to determine the attitudes of medical students and interns toward patient safety and the factors associated with it at a teaching hospital in Ernakulam, Kerala.

Methods: A cross-sectional study was undertaken among the final-year medical students and interns at a tertiary care teaching hospital in Kerala. Their attitude was assessed using the Attitude to Patient Safety Questionnaire III (APSQ III). The nine domains of the APSQ III were “patient safety training received, error reporting confidence, working hours as the cause of errors, error inevitability, professional incompetence as the cause of errors, disclosure responsibility, team functioning, patient involvement in reducing errors, and the importance of patient safety in the curriculum.” Convenience (non-probability) sampling was done, and all final-year medical students and interns were invited to participate in the study. Data were collected via Google Forms and were analyzed using IBM SPSS Statistics for Windows, Version 21.0 (Released 2012; IBM Corp., Armonk, NY). All scores have been reported as mean with SD, and univariate analysis was done using an independent samples t-test. The independent variables were the age of the participants, gender, and year level. The Institutional Ethics Committee provided ethical clearance for the study.

Results: A total of 144 students responded, of which 87 were female students (60.4%) and 82 (56.9%) were final-year Bachelor of Medicine and Bachelor of Surgery students. The overall score for the APSQ III was 5.31 ± 0.51, indicating an overall positive attitude. Six of the nine domains of the APSQ III reflected a positive attitude. The “working hours as the cause of errors” domain attained the highest mean score of 6.2, with a statistically significant difference between different age groups (p = 0.02). The domain “disclosure responsibility” showed a significant difference between genders (p = 0.02). The majority of the interns proposed an increased number of working hours to be the cause of medical errors (p = 0.001).

Conclusion: Though all students had an overall positive attitude toward patient safety, the domains “patient safety training received,” “professional incompetence as the cause of errors,” and “disclosure responsibility” were perceived with a negative attitude. Hence, a structured education program with assessments on patient safety training should be included in the medical curriculum to ensure that errors are minimized.

## Introduction

The World Health Organization defines patient safety as “the absence of preventable harm to a patient during the process of health care together with the reduction of risk of unnecessary harm associated with health care to an acceptable minimum” [1]. The likelihood of a patient experiencing harm during medical care is 1 in 300, compared to one in a million chance of being injured while flying. Many industries recognized for their high-risk environments actually maintain better safety records than the healthcare sector [2].

The most common medical errors include diagnostic errors, under- and overtreatment, surgical injuries, and adverse drug reactions, among others. Intensive care units, operating rooms, and emergency departments in hospitals are highly likely to be the sites of medical errors which could result in serious consequences [3]. Extremes of age, undergoing new procedures, and the urgency and seriousness of the procedure are associated with high error rates [3].

“To Err is Human,” a landmark report issued in November 1999 by the US Institute of Medicine, concluded that between 44,000 and 98,000 people die each year as a result of preventable medical errors [4]. It is estimated that around 5,000,000 deaths occur annually in India due to medical errors [5]. In the modern digital era, with the spread of social networking sites, medical errors easily grab public attention and thus affect doctor-patient relationships and trust.

Cultivating a strong attitude toward patient safety among medical students is crucial in minimizing these errors. Studies on patient safety from across the globe report mean scores ranging from 4.7 to 6.2, indicating a positive attitude among medical students [6-8]. However, a study done in Hong Kong indicated substantial deficiencies in medical students’ understanding of the constructive management of patient safety [9]. Understanding the importance of side-stepping from medical errors by having a preliminary knowledge of how to deal with the same is of utmost importance [10]. Medical errors occur as a culmination of several factors, including system factors. System solutions will therefore help to minimize errors in human cognition and memory. A study conducted on fourth-year medical students in Malaysia who had already undergone training on patient safety measures found that there was a definite positive correlation between the awareness of the safety of the healthcare system and personal influence over safety and attitudes to patient safety [11].

Many measures have been taken to assess medical errors and to ensure patient safety. Despite all the training and course implementation, the attitude of undergraduate medical students and interns remains a deciding factor in improving patient safety. Many questionnaires have been designed to examine the attitude of medical students toward patient safety, such as the Madigosky Questionnaire [12] and the Attitude to Patient Safety Questionnaire III (APSQ III) [13]. These tools help in understanding where the medical students stand with regard to patient safety, thereby taking the necessary steps to guide them through special courses or training programs [6].

In these circumstances, we have observed a lack of robust research on the topic of patient safety and the attitude of medical students toward it. Existing literature assesses the dimensions of patient safety education in varying situations, analyzing the differences with respect to the gender, prior experience with medical error, comparisons between students who took the patient safety curriculum and those who did not, the effects of low-income settings on students’ perception and awareness toward the issue, and many such varying scenarios worldwide. However, there is still a vast inadequacy of published studies in India. Therefore, the objective of this study was to assess the attitudes of medical students and interns toward patient safety at a teaching hospital in Ernakulam, Kochi.

## Materials and methods

A hospital-based cross-sectional survey was undertaken among final-year medical students and interns at Amrita Institute of Medical Sciences in Ernakulam, Kerala. Medical students who were currently not attending clinical postings due to any reason were excluded from the study. Convenience-based non-probability sampling technique was used as all the final-year students and interns were invited to take part in this research. Data were collected over a period of one month (July-August 2023) via Google Forms. Each respondent was allowed only a single response. The information regarding sociodemographic characteristics, patient safety training, and attitude toward patient safety was collected using a semistructured questionnaire.

Based on a study conducted by Alshahrani et al. [6] among medical students using the APSQ III questionnaire, the lowest observed mean score was for “error reporting confidence (4.7+ 1.56).” Applying this to the formula n = Z21-α/2 S2/d2, the minimum calculated sample size was 48, with a 95% confidence interval, 10% relative precision, and 10% dropout rate. 

The attitude toward patient safety was assessed using the APSQ III developed by Carruthers et al. [13]. The questionnaire contains 26 questions, which are divided into nine domains: patient safety training received, error reporting confidence, working hours as the cause of errors, error inevitability, professional incompetence as the cause of errors, disclosure responsibility, team functioning, patient involvement in reducing errors, and the importance of patient safety in the curriculum. The questions are scored on a 7-point Likert scale, with eight questions being reversely graded. A score greater than 4 is taken as a positive attitude, less than 4 as a negative attitude, and 4 as neutral.

Informed consent was taken prior to the administration of the questionnaire. The study was cleared by the Institutional Ethics Committee.

The data were analyzed using IBM SPSS Statistics for Windows, Version 21.0 (Released 2012; IBM Corp., Armonk, NY). Descriptive statistics such as frequency and percentage and mean and SD have been used to present the demographic data. The attitude toward patient safety has been displayed in mean ± SD.

Univariate analysis was done for patient safety with respect to the gender, year level, and age of the respondents. Independent samples t-test was done to test the association for variables following normal distribution (mean score ± SD) and the Mann-Whitney U test for non-normal data (median score with the difference of interquartile range (IQR)).

## Results

A total of 144 responses were collected from final-year medical students and interns. The response rate was 72%, of which 87 were female students (60.4%). Among the respondents, 63 (43.8%) were 23 years old, and the age ranged from 21 to 26 years. Six (4.2%) of the respondents were 21 and five (3.5%) were ≥25 years old. Of the respondents, 82 (56.9%) were final-year Bachelor of Medicine and Bachelor of Surgery (MBBS) students. Of the study population, 80 (55.6%) reported that they had not received any formal training on patient safety.

For all the questions on the nine domains of the APSQ III, grading was done on a 7-point Likert scale. A mean score greater than 4 was taken as a positive response, less than 4 as a negative response, and a perfect score of 4 as neutral.

The mean scores with SD are mentioned in Table 1 for all the nine domains of the APSQ III. The highest mean scores were observed for working hours as the cause of errors (6.2±0.98), followed by error inevitability (6.11 ±0.98), and team functioning (6.10±1.02), indicating a positive response in these domains.

**Table 1:** Distribution of mean scores for each domain of the APSQ III APSQ III: Attitude to Patient Safety Questionnaire III.

Domain	Mean ± SD	Positive response (%)
Patient safety training received	4.78 ± 1.36	71.5
Error reporting confidence	5.20 ± 1.34	78.5
Working hours as the cause of errors	6.2 ± 0.98	95.8
Error inevitability	6.11 ± 0.98	94.4
Professional incompetence as the cause of errors	4.43 ± 1.13	61.1
Disclosure responsibility	4.64 ± 1.19	62.5
Team functioning	6.10 ± 1.02	93.1
Patient involvement in reducing errors	5.61 ± 1.23	86.8
Importance of patient safety in the curriculum	5.23 ± 0.91	89.6

When the scores for each question in each domain were considered, the questions “If people paid more attention at work, medical errors would be avoided,” “Doctors have a responsibility to disclose errors to patients only if they result in patient harm,” and “Patient safety issues cannot be taught and can only be learned by clinical experience when qualified” were perceived with a negative attitude (Table 2).

**Table 2:** Distribution of mean scores for each question of the APSQ III domains APSQ III: Attitude to Patient Safety Questionnaire III. (R) implies that these questions were reverse-coded.

Domain	Question	Mean ± SD
Patient safety training received	My training is preparing me to understand the causes of medical errors.	4.95 ± 1.43
	I have a good understanding of patient safety issues as a result of my undergraduate medical training.	4.51 ± 1.50
	My training is preparing me to prevent medical errors.	4.88 ± 1.61
Error reporting confidence	I would feel comfortable reporting any errors I had made, no matter how serious the outcome had been for the patient.	5.27 ± 1.64
	I would feel comfortable reporting any errors other people had made, no matter how serious the outcome had been for the patient.	5.03 ± 1.51
	I am confident I could talk openly to my supervisor about an error I had made if it had resulted in potential or actual harm to my patient.	5.29 ± 1.60
Working hours as the cause of errors	Shorter shifts for doctors will reduce medical errors.	6.30 ± 1.10
	By not taking regular breaks during shifts, doctors are at an increased risk of making errors.	6.40 ± 1.06
	The number of hours doctors work increases the likelihood of making medical errors.	6.18 ± 1.26
Error inevitability	Even the most experienced and competent doctors make errors.	6.32 ± 0.99
	A true professional does not make mistakes or errors. (R)	5.84 ± 1.60
	Human error is inevitable.	6.16 ± 1.25
Professional incompetence as the cause of errors	Most medical errors result from careless nurses. (R)	5.03 ± 1.51
	If people paid more attention at work, medical errors would be avoided. (R)	3.23 ± 1.42
	Most medical errors result from careless doctors. (R)	4.72 ± 1.55
	Medical errors are a sign of incompetence. (R)	4.74 ± 1.65
Disclosure responsibility	It is not necessary to report errors which do not result in adverse outcomes for the patient. (R)	4.74 ± 1.82
	Doctors have a responsibility to disclose errors to patients only if they result in patient harm. (R)	3.81 ± 1.80
	All medical errors should be reported.	5.36 ± 1.66
Team functioning	Better multidisciplinary teamwork will reduce medical errors.	6.07 ± 1.14
	Teaching teamwork skills will reduce medical errors.	6.13 ± 1.11
Patient involvement in reducing errors	Patients have an important role in preventing medical errors.	5.41 ± 1.53
	Encouraging patients to be more involved in their care can help reduce the risk of medical errors occurring.	5.82 ± 1.33
Importance of patient safety in the curriculum	Teaching students about patient safety should be an important priority in medical student training.	6.31 ± 1.09
	Patient safety issues cannot be taught and can only be learned by clinical experience when qualified. (R)	3.16 ± 1.81
	Learning about patient safety issues before I qualify will enable me to become a more effective doctor.	6.23 ± 1.13

Univariate analysis was done for patient safety with respect to the gender, year level, and age of the participants (Tables 3-5). Independent samples t-test was done to test the association for variables following the normal distribution (mean score ± SD) and the Mann-Whitney U test for non-normal data (median score with the difference of IQR).

**Table 3:** Univariate analysis of APSQ III domains with respect to the gender (n = 144) APSQ III: Attitude to Patient Safety Questionnaire III. *Independent samples t-test was used for domains following normality. The mean score with standard deviation is given for these variables. For the remaining domains, the Mann-Whitney U Test for association was done, and the median score with difference of interquartile range is presented for these variables. For the domain working hours as the cause of errors, the median score was higher among the male participants compared to the female participants.

Domain	Male	Female	p-value
Patient safety training received	5 (1)	5 (2)	0.495
Error reporting confidence*	5 ± 1.45	5.33 ± 1.25	0.142
Working hours as the cause of errors	7 (1.17)	6.66 (1.34)	0.472
Error inevitability	6.33 (1.84)	6.66 (1.34)	0.277
Professional incompetence as the cause of errors*	4.38 ± 1.25	4.46 ± 1.05	0.655
Disclosure responsibility*	4.34 ± 1.40	4.83 ± 0.98	0.025
Team functioning	6 (1.5)	6.5 (1.5)	0.335
Patient involvement in reducing errors	6 (1.75)	5.5 (2)	0.844
Importance of patient safety in the curriculum	5 (1)	5.33 (1)	0.746

**Table 4:** Univariate analysis of the APSQ III domain with respect to the year level APSQ III: Attitude to Patient Safety Questionnaire III. *Independent samples t-test was used for domains following normality. The mean score with standard deviation is given for these variables. For the remaining domains, the Mann-Whitney U test for association was done, and the median score with difference of interquartile range is presented for these variables.

Domain	Interns	Final-year MBBS students	p-value
Patient safety training received	5 (1.5)	5 (1.84)	0.456
Error reporting confidence*	5.12 ± 1.52	5.26 ± 1.20	0.577
Working hours as the cause of errors	7 (0.34)	6.33 (1.67)	0
Error inevitability	6.66 (1.34)	6.33 (1.67)	0.307
Professional incompetence as the cause of errors*	4.40 ± 1.28	4.45 ± 1.01	0.785
Disclosure responsibility*	4.58 ± 1.13	4.68 ± 1.23	0.598
Team functioning	6.5 (1.5)	6.5 (1.5)	0.711
Patient involvement in reducing errors	6 (2)	5.75 (1.5)	0.404
Importance of patient safety in the curriculum	5.33 (1.34)	5 (1)	0.615

**Table 5:** Univariate analysis of the APSQ III domains with respect to age (n = 144) APSQ III: Attitude to Patient Safety Questionnaire III. *Independent samples t test was used for domains following normality. The mean score with standard deviation is given for these variables. For the remaining domains, the Mann-Whitney U test for association was done, and the median score with difference of interquartile range is presented for these variables.

Domain	Age in years		p-value
	<23	≥ 23	
Patient safety training received	5 (2)	5 (1.66)	0.945
Error reporting confidence*	5.20 ± 1.22	5.20 ± 1.39	0.988
Working hours as the cause of errors	6.33 (1.67)	7 (1)	0.019
Error inevitability	6.16 (1.42)	6.66 (1.67)	0.307
Professional incompetence as the cause of errors*	4.45 ± 0.94	4.42 ± 1.20	0.87
Disclosure responsibility*	4.64 ± 0.98	4.64 ± 1.27	0.992
Team functioning	6 (2)	6.5 (1.5)	0.515
Patient involvement in reducing errors	5.50 (1.5)	6 (2.13)	0.448
Importance of patient safety in the curriculum	5 (1.08)	5.16 (1.09)	0.508

On comparing the APSQ III scores with respect to the gender, the mean scores for error reporting confidence (5.33 ± 1.25), professional incompetence as the cause of errors (4.46 ± 1.05), and disclosure responsibility (4.83 ± 0.98) and the median scores for error inevitability (6.66 (1.34)), team functioning (6.5 (1.5)), and the importance of patient safety in the curriculum (5.33 (1)) were higher among the female participants compared to the male participants. Disclosure responsibility (p = 0.025) was the only variable with a statistically significant association with gender (Table 3).

While comparing the APSQ III scores with respect to the year level, the median scores for working hours as the cause of errors (7 (0.34)), error inevitability (6.66 (1.34)), patient involvement in reducing errors (6 (2)), and importance of patient safety in the curriculum (5.33 (1.34)) were found to be higher among the interns when compared to the final-year MBBS students. However, working hours as the cause of errors (7 (0.34), p < 0.001) was the only statistically associated variable when the year level was considered (Table 4). 

While comparing the median scores of APSQ III with respect to the age, the score for working hours as the cause of errors (7 (1)), error inevitability (6.66 (1.67)), team functioning (6.5 (1.5)), patient involvement in reducing errors (6 (2.13)), and the importance of patient safety in the curriculum (5.16 (1.09)) were higher among the respondents who were ≥23 years old compared to those who were <23 years old. The only significant association was between working hours as the cause of errors when the age of the respondents was considered (7 (1), p = 0.019) (Table 5).

## Discussion

The objective of this research was to assess the attitude of the final-year medical students and interns toward patient safety at a teaching hospital in Ernakulam. The majority of the study respondents had not received any formal training on patient safety during their course, and this was similar to the findings from the USA and Egypt [14,15]. Another study that was conducted on medical students in Singapore and Hong Kong suggested that no student had received any formal training on patient safety during their course [16]. This is a matter of concern and shows the limitations of the existing medical curricula.

Working hours as the cause of errors showed the highest score out of all domains in our study, and this is similar to the findings from a study in Singapore and Hong Kong [16]. A study conducted regarding the working hours of hospital staff showed that increased working hours resulted in an increased risk of causation of errors [17]. The positive attitude of medical students toward working hours as the cause of errors was higher among interns than among the final-year students and among students >23 years old. Many respondents correlate longer working hours to greater chances of making errors. This could be due to the fact that there is a general thought that medical errors are dependent on individuals. Awareness of other potential contributors in the commission of medical errors such as system failures or procedural intricacies should be brought out [16].

In the domain of error inevitability, higher scores were observed among female students and among interns, and our findings were similar to research from Saudi Arabia and Germany [6,18]. This shows that students agreed that human errors are inevitable and error commission does not reflect on a person’s professionalism, competency, or experience. Error reporting confidence was observed to be higher among female students and among interns, contradictory to the findings from Germany [18].

In our study, the team functioning domain showed a high score and a positive attitude among the respondents. This finding was found to be consistent with the findings in a study conducted in Saudi Arabia [6] and Pakistan [8], where they had the highest scores for team functioning. This emphasizes the importance of teamwork and its inclusion in the field of medicine. Institutions should adopt team-based activities and should work on improving communication within the team to effectively carry out providing quality patient care [19].

In our study, the participants agreed that the inclusion of patient safety training (5.61 ± 1.23) in the curriculum would be beneficial for them. Students from Saudi Arabia [6], Pakistan [8], Germany [18], Singapore, and Hong Kong [16] also reported the same.

Errors are inevitable, have a multifactorial role in causation, and do not reflect on the individuals’ professional incompetence [20]. Thus, the inclusion of patient safety training in undergraduate medical curricula would strengthen the abilities of students to deal with situations wisely in their profession [21,22].

In the disclosure responsibility domain, the majority of the participants agreed that all medical errors must be reported, which is similar to the results found in Saudi Arabia [6] and Germany [18]. In our study, it was observed that female students have a higher mean score for disclosure responsibility than male students. According to Mazor et. al., not disclosing one’s errors resulted in reduced trust and satisfaction [23]. The apprehension of being a victim of violence might cause them to hesitate to disclose errors to the patients or their families.

Limitations of the study

The cross-sectional nature of the study limits the ability of the results to draw causal inferences. All other limitations of a cross-sectional survey including recall bias, selection bias arising from non-probability sampling, volunteer-induced bias, and lack of generalizability are applicable to this study.

## Conclusions

This questionnaire-based study revealed that the majority of the medical students showed a positive attitude toward patient safety. However, the study also unveiled the misconceptions that students held regarding certain elements of patient safety. Lack of disclosure responsibility and notions that professional incompetence corresponds to error causation still persist among students. They also realize the importance of having proper training on patient safety issues and error reporting. Not having a formal patient safety course in the medical curriculum stands as a major setback for students. This is reflected in their attitudes regarding proper patient safety etiquette. 

A structured patient safety education program should be included in the undergraduate medical curriculum to improve the current situation. Further studies should be done to assess its level of effectiveness. An effective error reporting system should be established in institutions, and students should be encouraged to report errors without fail. A culture of taking responsibility for disclosing errors should be encouraged among medical graduates. Improving the working pattern and hours of duty among medical and paramedical staff would also help in minimizing medical errors.
